# Correction to: Severe Cardiomyopathy as the Isolated Presenting Feature in an Adult with Late‐Onset Pompe Disease: A Case Report

**DOI:** 10.1002/jmd2.12134

**Published:** 2020-11-09

Sunita Bijarnia‐Mahay should be added to the author list of Mori et al. ^1^. Sunita Bijarnia‐Mahay's affiliation is: Institute of Medical Genetics, Sir Ganga Ram Hospital, New Delhi, India.

The following text should also be added under “Contributions of Individual Authors”:

Sunita Bijarnia‐Mahay: Patient evaluation and management, and critical thinking about the diagnosis.
